# Nucleic Acid-Based Sensing Techniques for Diagnostics and Surveillance of Influenza

**DOI:** 10.3390/bios11020047

**Published:** 2021-02-12

**Authors:** Samantha J. Courtney, Zachary R. Stromberg, Jessica Z. Kubicek-Sutherland

**Affiliations:** Physical Chemistry and Applied Spectroscopy, Los Alamos National Laboratory, Chemistry Division, Los Alamos, NM 87545, USA; sjc@lanl.gov (S.J.C.); zrs@lanl.gov (Z.R.S.)

**Keywords:** influenza, viral diagnostics, surveillance, nucleic acid amplification test, direct detection, pandemic preparedness

## Abstract

Influenza virus poses a threat to global health by causing seasonal outbreaks as well as three pandemics in the 20th century. In humans, disease is primarily caused by influenza A and B viruses, while influenza C virus causes mild disease mostly in children. Influenza D is an emerging virus found in cattle and pigs. To mitigate the morbidity and mortality associated with influenza, rapid and accurate diagnostic tests need to be deployed. However, the high genetic diversity displayed by influenza viruses presents a challenge to the development of a robust diagnostic test. Nucleic acid-based tests are more accurate than rapid antigen tests for influenza and are therefore better candidates to be used in both diagnostic and surveillance applications. Here, we review various nucleic acid-based techniques that have been applied towards the detection of influenza viruses in order to evaluate their utility as both diagnostic and surveillance tools. We discuss both traditional as well as novel methods to detect influenza viruses by covering techniques that require nucleic acid amplification or direct detection of viral RNA as well as comparing advantages and limitations for each method. There has been substantial progress in the development of nucleic acid-based sensing techniques for the detection of influenza virus. However, there is still an urgent need for a rapid and reliable influenza diagnostic test that can be used at point-of-care in order to enhance responsiveness to both seasonal and pandemic influenza outbreaks.

## 1. Introduction

Respiratory viruses represent a public health threat in the form of both seasonal and pandemic outbreaks. Globally, the World Health Organization estimated that 290,000 to 650,000 deaths are associated with influenza infection each year [1]. Influenza is a zoonotic disease with several previous examples of emergence of novel variants that escape human immunity leading to significant morbidity and mortality [2]. Countermeasures for influenza require both an accurate clinical diagnostic tool and improved surveillance capabilities [3]. Prevention of influenza infection from vaccination can be effective if the strains included in the vaccine match the circulating virus population. However, antigenic shift and drift and inaccurate surveillance can render suboptimal vaccine protection [4]. Influenza treatment consists of administration of Food and Drug Administration approved antiviral drugs including Rapivab, Relenza, Tamiflu, and Xofluza, which should ideally occur within 24 to 48 h of symptom onset to be effective [5]. The evolution of drug resistant strains of influenza limit effective therapeutic options [6]. Rapid influenza diagnostics that can detect antiviral resistance can significantly increase treatment efficacy [7]. Diagnostic tests are mostly used to test symptomatic individuals while surveillance tools are used to detect the virus in animals and humans, often in asymptomatic individuals [8]. Diagnostic assays require high specificity to avoid false positives while surveillance assays require high sensitivity to avoid false negatives [9]. The requirements of diagnostic and surveillance assays are quite different; however, nucleic acid-based tests (NATs) can often satisfy sensitivity and specificity requirements of both [10]. Here, we consider the utility of currently available influenza nucleic acid detection technologies in order to identify potential gaps in our ability to respond to the next influenza pandemic. 

Influenza is an enveloped, single stranded negative sense RNA virus classified in the family *Orthomyxoviridae*. There are four types of influenza: A, B, C, and D which are classified by surface glycoproteins, number of RNA segments, and variations of the ribonucleoprotein complex (RNP) [11,12]. Influenza A virus (IAV) has a wide host range including humans, mammals, birds, swine, horses, and bats [13]. They can be further categorized by subtype based on the surface glycoproteins hemagglutinin (HA) and neuraminidase (NA) responsible for binding to host cell sialic acid-containing receptors and viral release from infected cells, respectively [14]. In contrast, influenza B viruses (IBV) are not divided into subtypes and are primarily restricted to humans, although a few cases have been reported in seals [15]. Influenza C (ICV) viruses cause mild disease in humans and have been predominantly isolated in camels, dogs, and swine [16,17,18]. Influenza D (IDV) is an emerging virus that is primarily associated with cattle and swine, but may have the potential to infect humans [12]. Clinical diagnostics for influenza have primarily focused on IAV and IBV; however, the ease of zoonotic transmission of influenza underlies the necessity to diagnose types C and D as well.

**Figure 1:**
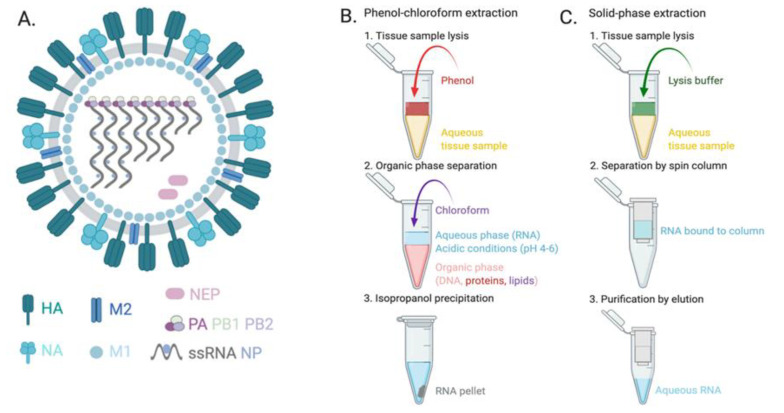
Detection of nucleic acids from influenza virus requires release from the intact virion. Viral RNA is encapsulated in (**A**) the influenza virion composed of a lipid and glycoprotein envelope containing single stranded RNA (ssRNA) and its associated proteins. The two most common sample processing methods used to extract RNA from the influenza virion include (**B**) phenol-chloroform and (**C**) solid-phase extraction by spin column. HA, hemagglutinin; NA, neuraminidase; M2; matrix ion channel; M1, matrix protein 1; NEP, nuclear export proteins; PA, PB1, PB2, RNA-dependent RNA polymerase subunits; NP, nucleoprotein. Figure created with Biorender.com.

The influenza virion is approximately 80 to 120 nm consisting of a membrane derived from host lipids incorporated with the ion channel protein M2 as well as the HA and NA glycoproteins (Figure 1A). Within this outer membrane is a layer of matrix protein 1 (M1). Inside the viral envelope are nuclear export proteins (NEP) and single stranded RNA (ssRNA) segments packaged by nucleoproteins (NP) and the RNA polymerase subunits (polymerase acidic (PA), RNA-directed RNA polymerase catalytic subunit (PB1), and polymerase basic protein 2 (PB2)). Influenza viruses possess seven (ICV and IDV) or eight RNA segments (IAV and IBV) [11,19]. In humans, the influenza virus replicates in epithelial cells of the nose, throat, and lungs [20]. The success of NATs relies on several key factors: (1) the virus must be in a high enough concentration and (2) nucleic acids must be liberated from the virion to be available for detection to occur.

The location and method of specimen collection is critical to ensure the highest possible viral load in the sample. Nasopharyngeal swabs (NPS) are the gold standard for upper respiratory tract specimen collection [21]. A significant limitation of this sample collection method is that it cannot be used on infants. As sensitivity of influenza molecular tests increases, less invasive sample types are being explored including nasal aspirates, nasal swabs, nasal washes, oropharyngeal (OP) swab, and saliva [22,23,24]. The various collection methods for detection of influenza virus have been reviewed by Spencer et al. showing that all specimen collection methods had high sensitivities (≥82%) in children [25]. In adults, larger differences in sensitivities were observed as a function of specimen type. A study by Lieberman et al. demonstrated that NP washes had higher sensitivities than either NP or OP swabs [23]. Another factor to sensitivity for adult specimens is time of collection post infection. In human volunteers experimentally infected intranasally with IAV, viral titers peaked at 2 days post infection [26]. After collection, the sample is processed to extract and stabilize the influenza RNA before detection [27]. There are several methods for extracting RNA, the most common being phenol-chloroform extraction (Figure 1B) and solid-phase extraction via spin column or magnetic beads (Figure 1C) [28]. Guanidinium isothiocyanate-phenol (also known as TRIzol) is widely used in phenol-chloroform RNA extraction procedures because it solubilizes nucleic acids while simultaneously denaturing proteins including RNases [29]. In recent years, commercialized kits and automated instrumentation for these extraction methods have been developed by various suppliers [30]. To streamline extraction further, there have been developments in microfluidic technology for both TRIzol and solid-phase methods [31]. After sample collection and liberation of the nucleic acids through sample processing, RNA can be detected using either amplification-based or direct detection techniques.

## 2. Amplification-Based Techniques

Nucleic acid amplification techniques are widely used for the diagnosis and detection of influenza [32,33]. Amplification is often needed to detect the presence of viral nucleic acid due to the low concentrations observed in patient samples [34]. There has been widespread adoption of traditional polymerase chain reaction (PCR)-based molecular assays. However, the equipment and reagent requirements of thermal cycling limit its point-of-care deployment. Therefore, more efficient isothermal amplification techniques have also been explored for on-site applications. Recent advances in traditional and isothermal amplification-based RT-PCR assays for detecting influenza have been summarized in Table 1.

### 2.1. Thermal Cycling Amplification Methods

#### 2.1.1. Real-Time Reverse Transcriptase PCR

The gold standard for influenza detection has shifted from traditional viral culture to real-time reverse transcriptase PCR (RT-PCR) [55]. Influenza viral RNA undergoes reverse transcription into complementary DNA (cDNA), then the target gene is amplified using specific DNA primers and DNA polymerase [56]. RT-PCR uses either intercalating dyes or fluorescently labeled probes, which are detected by the instrument. Intercalating dyes, such as SYBR green or methylene blue, emit a fluorescent signal upon binding double stranded DNA amplified in the reaction, which is cost-effective but lacks specificity that can lead to false positive results [57]. Fluorescent probes bind only specific DNA sequences but are more expensive. The most common probe formats used in influenza diagnostics are hydrolysis probes and molecular beacons (MBs) [58]. Hydrolysis probes contain a fluorophore and quencher which are separated through degradation of the probe during the amplification reaction. The advantages of hydrolysis probes are high specificity, reduced background fluorescence, and the ability to multiplex using various fluorophores [59]. MBs also contain a fluorophore and quencher which are separated by displacement (not degradation) during the amplification allowing for the same advantages as hydrolysis probes as well as the potential for allelic discrimination. However, MBs are difficult to design correctly due to their stringent physical requirements [60]. 

To decrease the time and cost associated with traditional RT-PCR, microwell PCR systems have been designed to decrease reagent volumes from 20 to 5 µL [36]. Multiplex assays have been designed to reduce costs by testing for the presence of multiple viruses or viral subtypes in a single sample [37]. Zhang et al. developed a sensitive multiplex assay for the simultaneous detection of all four influenza types (IAV, IBV, ICV, and IDV), which was validated on over 2000 animal samples [38]. However, commonly available fluorophores are not readily amenable to multiplexing due to their broad emission spectra and high background signals [61]. Further, diagnostic expenses increase with each additional probe and sensitivity is necessarily reduced due to the dilution of each probe per reaction [62]. 

#### 2.1.2. Microfluidic Automation of RT-PCR

Microfluidic technology has improved the efficiency of RT-PCR by reducing the reagent volumes and hands-on time for personnel. Microfluidics have also been explored as a means for assay automation that could support the use of RT-PCR at the POC [41]. RT-PCR microfluidic chips contain several channels that accommodate the various temperatures required for denaturation, annealing, and extension while the reaction undergoes continuous flow [63]. These advances have reduced the time to result in as little as 15 min; however, the assay is over 1000-fold less sensitive than traditional RT-PCR [39]. In another design, the RT-PCR reaction is contained within the microfluidic chip while a separate DNA detection kit must be used to quantify the results [41]. Influenza subtyping has also been accomplished using a microfluidic RT-PCR system utilizing glycan-coated magnetic beads [42]. 

### 2.2. Isothermal Amplification Methods

Isothermal amplification is performed at a single reaction temperature. When compared to methods that require thermal cycling, isothermal amplification is rapid, less expensive, and more energy efficient. These features would be advantageous for POC diagnostic devices, deployable instruments, and in resource-limited settings [64]. Isothermal strategies have been widely applied for detecting influenza.

#### 2.2.1. Loop-Mediated Isothermal Amplification (LAMP)

**Figure 2:**
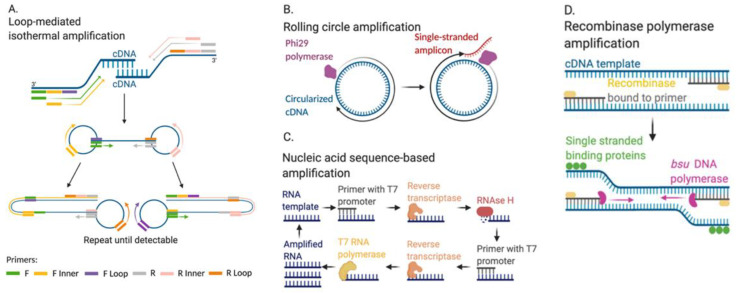
Isothermal amplification-based approaches to detection of influenza RNA. (**A**) Loop-mediated isothermal amplification (LAMP); (**B**) rolling circle amplification (RCA); (**C**) nucleic acid sequence-based amplification (NASBA); (**D**) recombinase polymerase amplification (RPA). F, forward; R, reverse. Figure created with Biorender.com.

LAMP requires outer, inner, and loop primer pairs designed for hybridization and amplification of a cDNA target sequence (Figure 2A). The outer and inner primer pairs amplify the double-stranded target sequence via self-hybridization within the newly amplified strands. With each new strand, this self-hybridization forms dumbbell-like shaped amplicons, introducing loop primer pair binding sites. With a minimum of six primer binding sites, the target sequence is exponentially and isothermally amplified. Both reverse transcriptase and DNA polymerase are included in the reaction mixture for amplification of influenza RNA viruses [65]. Similar to RT-PCR, amplified DNA concentrations can be quantified via intercalating dyes that emit a fluorescent signal. Influenza subtype-specific primers have been designed to target the HA gene across all known H1N1 strains [43]. These primers amplified IAV from human clinical samples in 40 min with a sensitivity of 97.8% and specificity of 100%. LAMP has also been multiplexed to accomplish both detection and influenza subtyping in 1 µL reaction volumes with solid state reagents [45]. Using six primer sets, this study amplified the H1 and H3 genes IAV and the NS1 gene from IBV in 30 min, with sensitivities of 96.4%, 100%, and 100% respectively. LAMP has also been used in a microfluidic device with a 90.90% sensitivity to detect IAV H1N1 in 30 min [46]. By maintaining isothermal conditions, LAMP is cost-effective, only requiring a heating block for amplification. However, primer design is a complex and time-consuming process that requires significant expertise.

#### 2.2.2. Rolling Circle Amplification

Rolling circle amplification (RCA) requires a specific DNA polymerase called Phi29. Target cDNA is converted into circular DNA through ligation of the 5′ and 3′ ends using DNA ligase. The specific primers then bind to the circular DNA template, and Phi29 DNA polymerase extends the target sequence in one continuous single DNA strand (Figure 2B). This single-stranded DNA amplicon contains hundreds of tandem repeats of the gene of interest and can be quantified with fluorescent probes or MBs within the one-step RCA reaction. This method is cost-effective because it does not require any instrumentation, but primer design is complex and most DNA quantification methods still require gel electrophoresis [66]. RCA has been utilized to detect IAV H1N1 in a colorimetric method using gold nanoparticles [47]. However, detection of an RNA template has not been shown. 

#### 2.2.3. Nucleic Acid Sequence-Based Amplification

Real-time nucleic acid sequence-based amplification (NASBA) amplifies multiple genes in a target RNA sequence using reverse transcriptase, RNaseH, and RNA polymerase (Figure 2C) [67]. NASBA requires a forward primer with a T7 promoter region that binds to a target RNA sequence, which enables reverse transcriptase to extend the target sequence. After extension, RNase breaks down the original RNA target sequence. A second primer binds to the new amplicon and extends that sequence via reverse transcriptase. T7 RNA polymerase binds to the extended amplicon, and RNA is synthesized. This process of reverse transcription, RNase activity, and RNA polymerase amplification is repeated until the RNA is detectable. For detection applications, MBs can be used to bind amplicons [68]. NASBA has been used to detect IAV H5N1 [49]. Simple amplification-based assay (SAMBA) is a NASBA based method that utilizes a nitrocellulose dipstick to visualize the test result. SAMBA has been used to detect IAV H1NI in 262 patient samples with sensitivity of 95.3% and specificity of 99.4% [51]. Once isothermal amplification was completed via NASBA, a dipstick was inserted into the reaction mixture for visualization of the signal with a total assay time of 85 min.

#### 2.2.4. Recombinase Polymerase Amplification and CRISPR-Based Diagnostics

Clustered regularly interspaced short palindromic repeats (CRISPR)-Cas (CRISPR-associated proteins) techniques have been applied recently to influenza detection. The function of CRISPR was first identified as a bacterial adaptive immune system against bacteriophages in 2007 [69]. More recently CRISPR-Cas systems have been developed for use in diagnostics. The most well-known systems include the DNA endonuclease-targeted CRISPR trans reporter (DETECTR) [70] and Specific High-Sensitivity Enzymatic Reporter UnLOCKing (SHERLOCK) [71]. The DETECTR system uses Cas12 to target DNA followed by indiscriminate single-stranded DNA reporter cleavage [70]. DETECTR has been applied to SARS-CoV-2 detection [72], but to our knowledge, it has not been expanded to influenza virus detection, while SHERLOCK exploits Cas13 nuclease activity that targets RNA and subsequently indiscriminately cleaves RNA reporters [71]. Although signal amplification can occur through recognition of a single sequence that in turn results in cleavage of multiple reporters, an amplification step is still needed for both methods.

For influenza detection, a combined SHERLOCK diagnostic approach with a therapy was developed, termed Cas13-Assisted Restriction of Viral Expression Readout (CARVER) as an “end-to-end” platform that demonstrated detection of IAV ssRNA as well as antiviral activity in less than 2 hours [52,73]. For amplification of target influenza RNA, SHERLOCK consists of isothermal recombinase polymerase amplification (RPA) followed by T7 polymerase transcription in vitro and detection of RNA by Cas13. RPA involves primers bound by recombinase proteins that target homologous sequences in the template strand, while single-stranded binding proteins keep the primers from dissociating from the template. Then, a displacement polymerase, most commonly bsu polymerase, amplifies the sequence isothermally (Figure 2D). After in vitro T7 transcription, target RNA and Cas13 are combined in a reaction where Cas13 cleaves fluorescent RNA cleavage reporters bound to the target. The signal is quantified via a plate-based fluorescence reader or a lateral flow strip test. This method is highly specific, so a single mismatch in the target sequence will escape detection. Another CRISPR-Cas13 assay termed Combinational Arrayed Reactions for Multiplexed Evaluation of Nucleic Acids-Cas13 (CARMEN-Cas13) was developed for simultaneous detection of 169 human viruses with attomolar sensitivity [54]. For the CARMEN-Cas13 method, RPA isothermal amplification is performed, and then, samples are mixed with nanoliter droplets containing detection reagents and loaded into a microwell-array chip where detection occurs. The CARMEN-Cas13 assay demonstrated IAV detection as well as viral subtyping for H1-H16 and N1-N9 [54]. These novel approaches, DETECTR, SHERLOCK, CARVER, and CARMEN, are promising platforms that could provide ultrasensitive influenza diagnosis and subtype discrimination.

## 3. Direct Nucleic Acid Detection Techniques

Direct detection of influenza RNA in clinical samples can enable rapid and accurate results without the need for nucleic acid amplification, which is time consuming and often requires expensive instruments and reagents [10]. Direct detection of nucleic acids is often performed through immobilization of an oligonucleotide probe on a substrate followed by hybridization with the target sequence resulting in a signal readout [74]. Immobilized oligonucleotides can consist of ssDNA or aptamers [75]. The detection signals for nucleic acid-based biosensors are often optical and electrochemical and can detect as low as 10^−18^ M target concentrations [74,76,77]. These techniques can provide rapid results with high throughput and excellent sensitivity. However, the equipment can be expensive, requires trained personnel, and reduces accuracy in complex biological samples [78]. Recent advances in techniques for the direct detection of influenza nucleic acids have been summarized in Table 2.

### 3.1. Optical Techniques

Optical biosensors have been widely developed for the direct detection of influenza nucleic acids with varying methodologies [78,91]. MBs have also been applied towards the amplification-free detection of influenza nucleic acids directly in human samples. MBs have been conjugated to near-infrared quintenary CdZnSeTeS quantum dots (QDs) to detect influenza RNA directly spiked into human serum [79]. This assay was completed in 3 min and detected as low as 1.9 copies viral RNA/mL, showing both effective QD signal amplification and MB hybridization to viral RNA in a complex human sample. However, the use of Cd-containing quantum dots presents hazards associated with handling heavy metals. In addition, MBs have been adapted to concurrently detect two separate genes fragments, specifically HA and NA, with only one MB [81]. This is made possible with the help of an assistant strand that binds to both gene fragments, allowing the MB to form a four-way junction with the assistant strand, NA gene fragment, and HA gene fragment for optical detection in 5 min with a detection limit of 120 pM.

Aptamers are oligonucleotide sequences composed of either DNA or RNA that are selected to bind to a target molecule [99]. Aptamers are selected using Systematic Evolution of Ligands by Exponential Enrichment (SELEX), which is an intensive iterative process of affinity selection starting with a diverse library of random oligonucleotides incubated with the molecule of interest [100]. Aptamers have been selected for use in optical biosensors to detect influenza using various techniques. DNA aptamers have been used to detect H1N1 viral RNA using QD fluorescence polarization in 95 min with a detection limit of 3.45 nM viral RNA [82]. Labeled DNA aptamers have also been used to detect avian influenza virus (AIV) H5N1 on a gold surface plasmon resonance (SPR) surface in 95 min with a detection limit of 0.128 hemagglutinating units (HAU) [85].

Fluorescence resonance energy transfer (FRET) has also been used to detect influenza nucleic acids [83]. A CdSe quantum dot-labeled single-stranded DNA probe immobilized on a carbon nanotube (CNT) was shown to detect 9.39 nM target viral DNA although RNA was not tested and detection in complex biological samples was not performed.

Peptide nucleic acids (PNA) have been used with gold nanoparticles as another biosensor application for optical detection of influenza RNA [86]. In this simple assay, non-hybridized PNAs induced gold nanoparticle aggregation, and PNAs hybridized to RNA did not induce aggregation. This colorimetric, spectrophotometer-based detection was completed in 11 min with a detection limit of 2.3 ng RNA. This detection was validated using influenza RNA extracted from clinical samples.

### 3.2. Electrochemical Techniques

Electrochemical sensors are promising tools to detect influenza nucleic acids directly without amplification due to their rapid, sensitive and low-cost instrumentation [78]. The electrochemical detection of influenza nucleic acids has been performed using CNT based field effect transistors where a single-stranded DNA probe was immobilized in a CNT field effect transistor channel and then submerged into a solution of single-stranded target DNA [87]. Upon probe-target hybridization, the current in the CNTs decreases and within 1 min detects as little as 1 pM target DNA. However, the change in current could not be directly attributed to DNA hybridization since the test was not performed with non-complementary target DNA for comparison. Another electrochemical detection platform utilized electrochemical impedance spectroscopy (EIS) to detect influenza target DNA immobilized on a CNT substrate [88]. A single-stranded DNA probe was immobilized on a CNT electrode then incubated with biotin-labeled target oligonucleotide followed by exposure to streptavidin-labeled gold nanoparticles to amplify the signal. Influenza detection was performed in 35 min with as little as 557 pM target DNA. In another CNT application, multi-walled nanotubes (MWCNTs) were able to detect IAV in 4 min with a detection limit of 0.5 nM [89]. CNTs have also been altered to binary-nanoparticle-decorated nanotubes (bNP-CNTs) for influenza detection. In an application of this hybrid nanomaterial, the bNP-CNTs were synthesized as gold/iron-oxide decorated CNTs on an interdigitated surface. The decorated CNT platform was able to immobilize a DNA probe and detect influenza virus down to 8.4 pM [90]. Although promising, none of these CNT-based electrochemical techniques were tested directly on influenza RNA or in any clinical sample matrices. 

**Figure 3:**
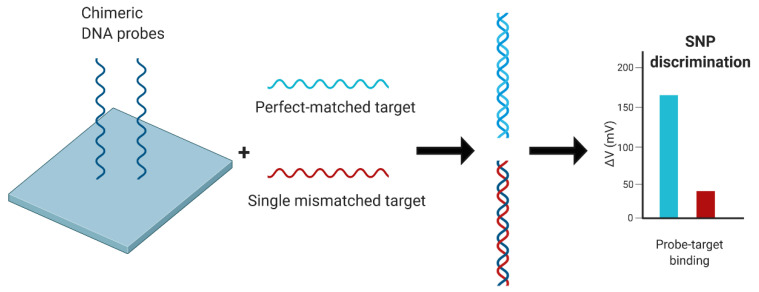
Schematic of the chimeric DNA probe and target binding on a surface plasmon resonance (SPR) device for SNP detection as described previously [92]. Figure created with Biorender.com.

DNA probes have been applied to several gold electrochemical surfaces. On a gold SPR surface, chimeric DNA probes were immobilized to the surface and were able to simultaneously hybridize to an influenza H1 DNA target and detect single nucleotide polymorphisms (SNPs) [91] (Figure 3). On another gold electrode surface, two DNA probes were immobilized: one encoding for HA and another methylene blue-labeled probe encoding for NA. Together on the same surface, target DNA hybridized to both probes, which induced redox activity for detection via voltammetric signal at a limit of 8–100 nM [93]. In another application of a biosensor with a gold electrode surface, tetrahedral nanostructure DNA probes were immobilized via self-assembly to detect the influenza H7N9 HA gene via amperometric signal with a detection limit of 100 fM [94]. An electrochemical genosensor took advantage of a gold electrode by using it to create a redox active layer on which a DNA probe could bind via the probe amine group and redox layer epoxide group. With this specific amine-epoxy covalent reaction on the biosensor surface, the DNA probe was able to selectively detect IAV RNA with a detection limit of 73 pM [96]. Again, these gold-functioned electrochemical methods are promising due to their highly sensitive and specific capabilities, but were not tested on any clinical samples.

### 3.3. Next-Generation Sequencing

Next-Generation Sequencing (NGS) enables viral detection and characterization from a single sample [101]. NGS allows for the identification of genetic variants as well as unknown viruses [102]. However, NGS runs are time-consuming, instruments are expensive, and extensive bioinformatic analysis is required. The low amount of viral RNA present in human samples severely limits the efficacy of NGS. Target-based enrichment probes have been designed for upper respiratory viruses, including influenza [102]. Universal influenza primers have also been designed for IAV, IBV, and ICV [97]. In this previous study, the universal influenza RT-PCR assay produced large amplicons that were analyzed bioinformatically to determine the influenza sequences present in clinical samples. Other studies have used RNA sequencing to determine whether patients were infected with influenza [103,104]. This unbiased approach allows for screening of several respiratory pathogens that can often cause overlapping symptoms, thus making it difficult for clinicians to determine the etiological agent [105].

## 4. CLIA-Waived Nucleic Acid Diagnostics

Although nucleic acid testing for influenza has its advantages, it is important to recognize that these tests are only clinically relevant if introduced into a POC setting for diagnosis. Several RT-PCR instruments accompanied by their respective RT-PCR reagent kits were approved for POC operation in 2015, deemed by the U.S. Clinical Laboratory Improvement Amendments of 1988 (CLIA). CLIA regulates United States laboratory facilities that test human specimens for diagnosis, prevention, and treatment of disease. CLIA-waived tests are categorized by the following criteria [106]:

1. Minimal scientific knowledge for performance of test;
2. Minimal training and experience for performance and analysis of test;
3. Stable and reliable reagents (prepackaged, no special handling, room temperature storage conditions);
4. Automated or easily controlled operational steps;
5. Stable and readily available materials for calibration, quality control, and external proficiency;
6. Automated and/or easily performed troubleshooting and maintenance;
7. Minimal interpretation of results.

Nucleic acid detection based CLIA-waived tests for IAV, IBV, and/or respiratory syncytial virus (RSV) include cobas Liat Influenza A/B Assay, cobas Liat Influenza A/B & RSV Assay, ID NOW Influenza A & B 2, Xpert Xpress Flu, Xpert Xpress Flu/RSV, Silaris Influenza A & B Test, and BioFire FilmArray Respiratory (RP) EZ Panel. Each of these assays strives for high sensitivity and specificity, rapid performance time, and facilitating accurate clinical diagnosis.

Roche Diagnostics has released two influenza assays using the cobas Liat PCR System instrument--cobas Liat Influenza A/B Assay and cobas Liat Influenza A/B & RSV Assay. For both assays, a NPS is required as the patient sample. The cobas Liat PCR System performs RT-PCR in 20 min, and displays amplification curves and cycle thresholds (C_T_) [107,108]. This system allows for a small footprint and easy transfer to any laboratory or POC setting, and the assays provide minimal labor with a walk-away workflow. The reagents, however, must be refrigerated, which might be a restriction for some POC settings [109]. The instrument is USD 25,000, and each assay is around USD 72 per test. Overall, for the Influenza A/B Assay, this system provides a sensitivity of 100% and 94.4% to IAV and IBV, respectively, and a specificity of 98.3% and 100% to Influenza A and B, respectively [107]. For the Influenza A/B & RSV Assay, the system provides a sensitivity of 99.6% and 99.3% to IAV and IBV, respectively, and a specificity of 97.5% and 9.7% to IAV and IBV, respectively [108].

Abbott manufactures the ID NOW Influenza A & B 2 assay on the ID NOW platform, previously known as Alere i. This system performs an isothermal nucleic acid amplification test (NAAT) via a nicking enzyme and requires a NPS, direct nasal swab (NS), or both NPS and NS in viral transport medium (VTM) as the patient sample [108]. There are several advantages to this platform, including performance time, where positive results are displayed in 5 min, room temperature reagent storage, and small instrument size [107]. However, compared to other CLIA-waived influenza nucleic acid tests, there are several more workflow steps [109]. The price per assay is USD 105. Overall, for the Influenza A & B 2 assay, the ID NOW platform provides a sensitivity of 93.2% and 97.2% to IAV and IBV, respectively, and a specificity of 97% to both.

Cepheid manufactures the Xpert Xpress Flu and Xpert Xpress Flu/RSV assays, which are performed on the GeneXpert Xpress II and IV system. The GeneXpert Xpress performs RT-PCR in 30 min and requires either an NPS, nasopharyngeal aspirate (NA), or nasal wash (NW) as the patient sample. If there is a positive result within 20 min, this system has an option to bypass the negative control confirmation and terminate the assay early. As another advantage, the reagents are stored at room temperature. However, there are several disadvantages of this system compared to the cobas Liat and ID NOW platforms, including the larger amount of benchtop space required, as well as a higher rate of false negatives [108,109]. The instrument is USD 49,000 and each assay is around USD 55 per test. Overall, for the Xpert Xpress Flu assay, this system provides a sensitivity of 100% and 97.8% to IAV and IBV, respectively, and a specificity of 99.3–100% to both [108]. For the Xpert Xpress Flu/RSV assay, the system provides a sensitivity of 100% and 96.3% to IAV and IBV, respectively, and a specificity of 100% to both [110].

There are several comparison studies of cobas Liat ID NOW, and Xpert Xpress platforms [107,108,109,111,112]. Silaris Influenza A & B Test and BioFire FilmArray Respiratory EZ Panel, however, have not yet been compared in a published research study and the available information about these assays are from the manufacturer. Therefore, as of date, these platforms cannot yet be compared to other CLIA-waived methods in this review apart from the manufacturer’s listed specifications. Silaris Influenza A & B Test is manufactured by Mesa Biotech as Accula Flu A/Flu and distributed by Sekisui Diagnostics as Silaris in the United States. With a direct NS patient sample, the Silaris dock performs RT-PCR in 30 min with colorimetric visualization on a test stick. The dock and test are affordable (USD 180 per dock, USD 30 per test), maintenance-free, and reagents are stored at room temperature. Overall, for the Influenza A & B Test, the Silaris dock provides a sensitivity of 97% and 94% to IAV and IBV, respectively, and a specificity of 94% and 99% to IAV and IBV, respectively. BioFire Diagnostics manufactures the BioFire Film Array RP EZ, which performs multiplex nested PCR with a NPS patient sample in 60 min, paneling 14 respiratory pathogens in one assay. BioFire has not yet publicly released information about the sensitivity, specificity, and price of the instrument or assays.

When comparing all CLIA-waived nucleic acid detection methods, they are all highly sensitive and rapid (Table 3). Furthermore, Kanwar et al. claimed that cobas Liat, ID NOW, and Xpert Xpress platforms are comparable in sensitivity, specificity, ease of use, and short turnaround time using the CDC Flu A/B PCR assay for reference [109]. With the addition of Silaris Influenza A & B Test and BioFire FilmArray Respiratory EZ Panel, there are now seven CLIA-waived nucleic acid detection platforms for influenza. Since 2015, these platforms have been widely distributed in clinical laboratories and POC settings, yet several improvements are still needed. With those improvements in mind, many experimental detection methods previously discussed are attempting to bridge the gap between POC setting limitations and ultrasensitivity.

## 5. Conclusions and Future Directions

In summary, patient outcome of influenza infection is tied to rapid and accurate diagnostics. Rapid tests are often performed using antigen detection techniques which have been shown to display poor accuracy. Nucleic acid detection techniques are much more accurate but are traditionally time-consuming and expensive requiring trained personnel to perform the test. Here we have provided an overview of recent advances towards bringing nucleic acid detection of influenza closer towards the ultimate goal of a rapid, accurate, cost-effective detection platform designed for a POC setting. Nucleic acid amplification is still a requirement of the CLIA-waived diagnostic tests currently available, which increases the time, expense and reagent requirements of these tests. Ideally an influenza test used for both diagnostic and surveillance purposes would be faster and less expensive than those currently available in order to support continuous high-throughput screening of a high number of samples. 

Techniques that directly detect influenza nucleic acids in complex biological samples are a promising solution to achieve both rapid and ultrasensitive measurements for diagnostic and surveillance applications. A variety of optical and electrochemical techniques have been explored to detect influenza nucleic acids without amplification in as little as 1 min at pM detection limits; however, few studies have tested clinical samples, so it is not clear if these detection limits are low enough to be clinically relevant. Both optical and electrochemical techniques are highly sensitive to background noise, which makes testing directly in clinical samples very challenging and often requiring highly purified nucleic acids for testing. The sample purification process required for direct detection is very similar to that of amplification-based techniques. Further, many of these direct sensing techniques have used DNA as the substrate for their assays indicating the requirement of reverse transcription, so the ability of these sensors to directly detect influenza RNA in clinical samples requires further investigation. Additional optimization is required to simplify direct detection of influenza nucleic acids in a POC setting to enhance responsiveness to both seasonal and pandemic influenza outbreaks.

## Figures and Tables

**Table 1:** Comparison of amplification-based techniques for detecting influenza viruses.

Method	Influenza Type (Subtype)	Target Gene	Time to Result (min)	No. of Clinical Samples Tested	RNA Detection Limit	Advantages	Current Limitations	Ref.
**Thermal Cycling**								
CDC human influenza virus real-time RT-PCR diagnostic panel influenza A/B typing kit	A, B	M1, M2, NS1	~240	n.s.	n.s.	Several kit configurations to detect emerging strains, works on a variety of sample types	Available only to qualified DoD laboratories, U.S. public health laboratories, and NREVSS collaborating laboratories	[35]
Microwell array-based digital PCR	A (H7N9)	HA	60	None	700 copies/μL	Simple fabrication, user friendly operation, low device cost (<USD 1/unit)	High background signal caused by reporter dye, dust and other contaminants associated with PDMS material	[36]
Multi-fluorescent RT-PCR	A (H1N1)	HA, M1, M2	100	27 human	n.s.	High sequence coverage, improved multiplex performance	High specificity can lead to primers/probe failure upon viral mutation	[37]
Multiplex RT-PCR	A, B, C, D	M, PA, PB	60	758 swine and 1525 bovine	30 copies per 20 µL reaction (1.5 copies/µL)	Supports surveillance and diagnostics, high sequence coverage (98–100%), simultaneous detection of all 4 influenza types	IBV assays not yet validated in clinical samples	[38]
Microfluidic RT-PCR chip and electrochemical DNA sensor	A (H1N1)	M1	15	None	5 × 10^3^ copies/µL	Rapid, potentially portable and low cost	Poor sensitivity, requires validation in clinical samples	[39]
Microfluidic preconcentration and nucleic amplification system	A (H1N1)	M1	>120	None	100 TCID_50_	Pre-concentration and amplification in the same device, portable, low cost	Difficult fabrication process, requires validation in clinical samples	[40]
Microfluidic RT-PCR chip	A	M1	>120	146 human	1 copy/µL	Nucleic acid extraction and RT-PCR performed in a single chip, could support POC testing	Long time to result, requires further automation and optimization to achieve POC use	[41]
Microfluidic HA x NA subtyping array	A	HA, NA	100	None	~40 copies per 10 µL reaction (4 copies/µL)	Supports simultaneous diagnosis and subtyping, fully automated for POC testing	Variability in various subtyping assays, requires validation in clinical samples	[42]
**Isothermal**								
Reverse transcription-loop-mediated isothermal amplification (RT-LAMP)	A (H1N1)	HA	40	260 human	10 copies/25 μL (0.4 copies/µL)	Low-cost equipment and minimally trained personnel, well suited for resource-limited settings	2.2% false negative rate	[43]
Colorimetric RT-LAMP	A, B	HA, NA	60	135 human	0.1–100 genome copies	Reduced detection time compared to conventional PCR, does not require trained personnel	Not as accurate as qRT-PCR, complex primer design	[44]
Multiplex RT-LAMP	A (H1N3), B	HA, NS1	30	202 human	1 genome equivalent/25 µL reaction (0.04 copies/µL)	Ultra-sensitive, rapid 10 min mechanical sample processing method, rapid 12 min assay run time	3.6% false negative rate	[45]
Microfluidic LAMP	A	HA	30	77 human	n.s.	Rapid, low reaction volume of 1 µL, incorporates solid state reagents	Poor sensitivity (90.9%)	[46]
Rolling circle amplification (RCA)	A (H1N1)	M1, M2	>120	None	n.s.	Low-cost equipment, colorimetric result for ease of use	No reverse-transcriptase component, assay only tested with DNA template	[47]
RCA on polymer chip	B	n.s.	60	None	10 pM	Automated multi-step assay, potential POC application	Requires validation in clinical samples, complex chip design	[48]
Real time-nucleic acid sequence-based amplification (NASBA)	A (H5N1)	HA, NA	>120	19 human	10 copies/µL	Supports limited subtyping	Long time to result, recommended for use as a second line test	[49]
RT-NASBA	A (H1N1)	HA	90	67 human	3 copies/µL	No cross-reactivity, 100% sensitivity, speificity, and positive predictive value	Designed for detection of only one viral strain, limited sample comparison	[50]
Simple amplification-based assay (SAMBA)	A (H1N1)	HA, M1, M2	85	262 human	0.25 PFU per test	Low-cost equipment, easy to read result	Poor sensitivity (95.3%)	[51]
Cas13-assisted restriction of viral expression readout (CARVER)	A	NP, M1	<120	None	n.s.	End to end platform, multiplexed, Cas13 targeting does not lead to mutations	Needs to be validated on clinical samples	[52]
Real-time recombinase polymerase amplifcation CRISPR-Cas12a	A, B	M1	60	83 human	~10^2^ copies/µL	Multiplexed assay that can detect COVID-19 and influenza,	Detection of DNA	[53]
Combinatorial arrayed reactions for multiplexed evaluation of nucleic acids-Cas13 (CARMEN)	A	HA, NA	60	HIV clinical samples	aM level	Highly multiplexed, high sensitivity, low reagent cost (comparative to other SHERLOCK-based methods)	Complex primer and assay design, needs to be validated on influenza clinical samples	[54]

DoD, Department of Defense; NREVSS, National Respiratory Enteric Virus Surveillance System; HA, hemagglutinin; NA, neuraminidase; M1, matrix protein 1; M2, matrix ion channel; M, influenza C matrix protein; PA, polymerase acidic protein; PB, RNA-dependent RNA polymerase catalytic subunit; NS1, nonstructural protein 1; PDMS, polydimethylsiloxane; POC, point-of-care; NP, nucleoprotein; HIV, human immunodeficiency virus; SHERLOCK, specific high-sensitivity enzymatic reporter unlocking; n.s., not specified.

**Table 2:** Comparison of direct nucleic acid detection techniques for influenza viruses.

Method	Influenza Type (Subtype)	Target Gene	Time to Result (min)	RNA Detection Limit	Advantages	Current Limitations	Ref.
**Optical**							
NIR QD-molecular beacon bioprobe	A (H1N1)	NA	3	1.9 copies/mL	Works in complex biological samples	Hazards associated with Cd-containing QDs, requires validation in clinical samples	[79]
Activatable silver nanoclusters molecular beacon	A (H1N1, H5N1)	HA, NA	30	2 nM	One-pot detection of multiplex DNA, signal detection without fluorophore cleavage from quencher	Complex multiplexed beacon design, requires validation in clinical samples	[80]
Dual target molecular beacon sensor	A (H5N2)	HA, NA	5	120 pM	Highly specific concurrent multisequence detection, works in complex biological samples	Complex molecular beacon and assistant strand design, requires validation in clinical samples	[81]
QD fluorescence polarization probes with protein-binding aptamer signal amplification	A (H1N1)	n.s.	95	3.45 nM	Long shelf life, low-cost assay	Hazards associated with Cd-containing QDs, time-consuming, requires validation in clinical samples	[82]
QD to CNT FRET-based DNA	A (H5N1)	PB2	>120	9.39 nM	Low-cost assay	Long time to result, hazards associated with Cd-containing QDs, assay only tested with DNA template, requires validation in clinical samples	[83]
QD probe with custom-made portable sensor	A (H5N1)	HA	>120	12.5 µM	Simple design, portable, does not require wash steps	Result reading device does not have high sensitivity	[84]
SPR aptasensor	A (H5N1)	HA	95	0.128 HAU	Portable, validated by poultry swab samples	Expensive and complex assay design	[85]
Peptide nucleic acid biosensor	A	M1	11	2.3 ng	High specificity, label free optical detection, low cost, validated by RNA extracted from clinical samples	Complex design and characterization of peptide nucleic acids	[86]
**Electrochemical**							
CNT field effect transistor-based DNA sensor	A	M1	1	1 pM	Long shelf life	Assay only tested with DNA template, requires validation in clinical samples	[87]
Impedimetric DNA sensor using CNT and gold nanoparticle amplification	A (H1N1)	n.s.	35	557 pM	High sensitivity	Equipment operation and data analysis requires extensive training, assay only tested with DNA template, requires validation in clinical samples	[88]
Multi-wall CNT DNA sensor	A	M1, M2	4	0.5 nM	Low cost, interdigitated array microelectrode simplicity	Data analysis requires extensive training, assay only tested with DNA template, requires validation in clinical samples	[89]
Gold/iron-oxide CNT-hybrid nanomaterial DNA sensor	A (H1N1)	NA	n.s.	8.4 pM	High selectivity, simple apparatus	Hybrid nanomaterials are relatively uncommon for sensors and thus need further investigation, assay only tested with DNA template, requires validation in clinical samples	[90]
SPR biosensor for SNP DNA probe	A (H1)	HA	>120	n.s.	Highly stable probe, SNP descrimination	Sensor acquisition is time consuming, instrument operation and data analysis requires extensive training and costs, requires validation in clinical samples	[91]
RNase facilitated SPR detection of microRNA	A (H1N1)	MicroRNA 29a–3p	60	1 nM	Does not require thermal cycling, rapid reporter assay for microRNA, tested in clinical samples	Expensive instrumentation, low abundance of microRNA in diagnostic samples	[92]
Duo-genosensor with DNA probes	A (H5N1)	HA, NA	>120	8–100 nM	Two DNA probes lowers possibility of false positive readings, low cost to handle genosensor	Electrode preparation is time consuming (>18 h), expensive instrumentation, requires validation in clinical samples	[93]
DNA tetrahedral nanostructure-based biosensor	A (H7N9)	HA	n.s.	100 fM	Inexpensive equipment and operation, apparatus portable	High complexity tetrahedral probe design, requires validation in clinical samples	[94]
Microfluidic chip integrated with reduced graphene oxide transistor	A (H5N1)	HA	n.s.	5 pM	Flow through strategy provides sensitivity and stability	rGO transistors are not well studied, requires validation in clinical samples, time consuming impedance spectrum measurements	[95]
Redox-active genosensor	A (H5N1)	HA	n.s.	73 pM	High selectivity for RNA sequences	Not as sensitive to DNA sequences compared to RNA, requires validation in clinical samples	[96]
**Next-Generation Sequencing**							
Next-generation sequencing-based diagnostic	A, B	Full genome	>120	n.s.	Supports surveillance, diagnostics as well as simultaneous virulence and drug resistance profiling	Long time to result, extensive data processing required	[97]
Metagenomic Nanopore sequencing	A, B	Full genome	>120	10^2^–10^3^ copies/mL	Tested in 27 clinical samples, high speicificity (100%)	Low sensitvity (83%), long time to results, extensive data processing required	[98]

NIR, near-infrared; QD, quantum dot; CNT, carbon nanotube; FRET, fluorescence resonance energy transfer; SPR, surface plasmon resonance; SNP, single nucleotide polymorphism; HAU, hemagglutinating units; NA, neuraminidase; HA, hemagglutinin; M1, matrix protein 1; PB2, RNA-dependent RNA polymerase subunit; M2, matrix ion channel; n.s., not specified.

**Table 3:** Comparison of U.S. Clinical Laboratory Improvement Amendments of 1988 (CLIA)-waived diagnostic tests for influenza virus.

Kit	Manufacturer	Instrument	Method	Specimens ^1^	Assay Time (min)	Cost per Test	Sensitivity (Influenza A, B)	Specificity (Influenza A, B)	Ref.
BioFire FilmArray RP EZ	BioFire Diagnostics, Salt Lake City, UT, USA	FilmArray 2.0	Multiplex nested PCR	NPS	60	n.s.	N/A	N/A	Kit instructions
cobas Liat influenza A/B	Roche, Branchburg, NJ, USA	cobas Liat system	Real-time RT-PCR	NPS	20	USD 72.10	100%, 94.4%	98.3%, 100%	[107]
cobas Liat influenza A/B & RSV	Roche, Branchburg, NJ, USA	cobas Liat system	Real-time RT-PCR	NPS	20	USD 77.25	99.6%, 99.3%	97.5%, 99.7%	[108]
ID NOW Influenza A & B 2	Abbott, Lake Bluff, IL, USA	ID NOW Platform	Nicking enzyme isothermal NAAT	NPS, NS direct or in VTM	15	USD 105	93.2%, 97.2%	97%	[109]
Silaris Influenza A & B	Mesa Biotech, Inc., San Diego, CA, USA	Silaris Dock	RT-PCR	NS direct	30	USD 30	97%, 94%	94%, 99%	Kit instructions
Xpert Xpress Flu	Cepheid, Sunnyvale, CA, USA	GeneXpert systems GXII and GXIV	Real-time RT-PCR	NPS, NA, NW	30	USD 54.60	100%, 97.8%	99.3–100%	[108]
Xpert Xpress Flu/RSV	Cepheid, Sunnyvale, CA, USA	GeneXpert systems GXII and GXIV	Real-time RT-PCR	NPS, NA, NW	30	USD 86.50	100%, 96.3%	100%	[110]

^1^ NA, nasal aspirate; NPS, nasopharyngeal swab; NS, nasal swab; NW, nasal wash; VTM, viral transport medium; n.s., not specified.

## Data Availability

All data reviewed in this manuscript were obtained from published studies and can be obtained by accessing the reference cited for each data set.

## Abbreviations

Avian influenza virus (AIV); binary-nanoparticle-decorated nanotubes (bNP-CNTs); carbon nanotube (CNT); Cas13-assisted restriction of viral expression readout (CARVER); clinical laboratory improvement amendments (CLIA); clustered regulatory interspaced short palindromic repeats (CRISPR); combinatorial arrayed reactions for multiplexed evaluation of nucleic acids (CARMEN); complementary DNA (cDNA); CRISPR-associated proteins (Cas); cycle threshold (C_T_); DNA endonuclease-targeted CRISPR trans reporter (DETECTR); electrochemical impedance spectroscopy (EIS); fluorescence resonance energy transfer (FRET); hemagglutinin (HA); hemagglutinin units (HAU); influenza A virus (IAV); influenza B virus (IBV); influenza C virus (ICV); influenza D virus (IDV); loop-mediated isothermal amplification (LAMP); matrix ion channel (M2); matrix protein 1 (M1); molecular beacons (MBs); multi-walled nanotubes (MWCNTs); nasal swab (NS); nasal wash (NW); nasopharyngeal swab (NPS); nasopharyngeal aspirate (NA); near-infrared (NIR); neuraminidase (NA); next-generation sequencing (NGS); nuclear export proteins (NEP); nucleic acid amplification test (NAAT); nucleic acid-based tests (NATs); nucleic acid sequence-based amplification (NASBA); nucleoproteins (NP); oropharyngeal (OP); peptide nucleic acids (PNA); polymerase acidic (PA); polymerase basic protein 2 (PB2); polymerase chain reaction (PCR); quantum dot (QD); real-time reverse transcriptase PCR (RT-PCR); recombinase polymerase amplification (RPA); respiratory syncytial virus (RSV); reverse transcription-loop-mediated isothermal amplification (RT-LAMP); ribonucleoprotein complex (RNP); RNA-directed RNA polymerase catalytic subunit (PB1); rolling circle amplification (RCA); simple amplification-based assay (SAMBA); single nucleotide polymorphism (SNP); single stranded RNA (ssRNA); specific high-sensitivity enzymatic reporter UnLOCKing (SHERLOCK); surface plasmon resonance (SPR); systematic evolution of ligands by exponential enrichment (SELEX); viral transport medium (VTM).
